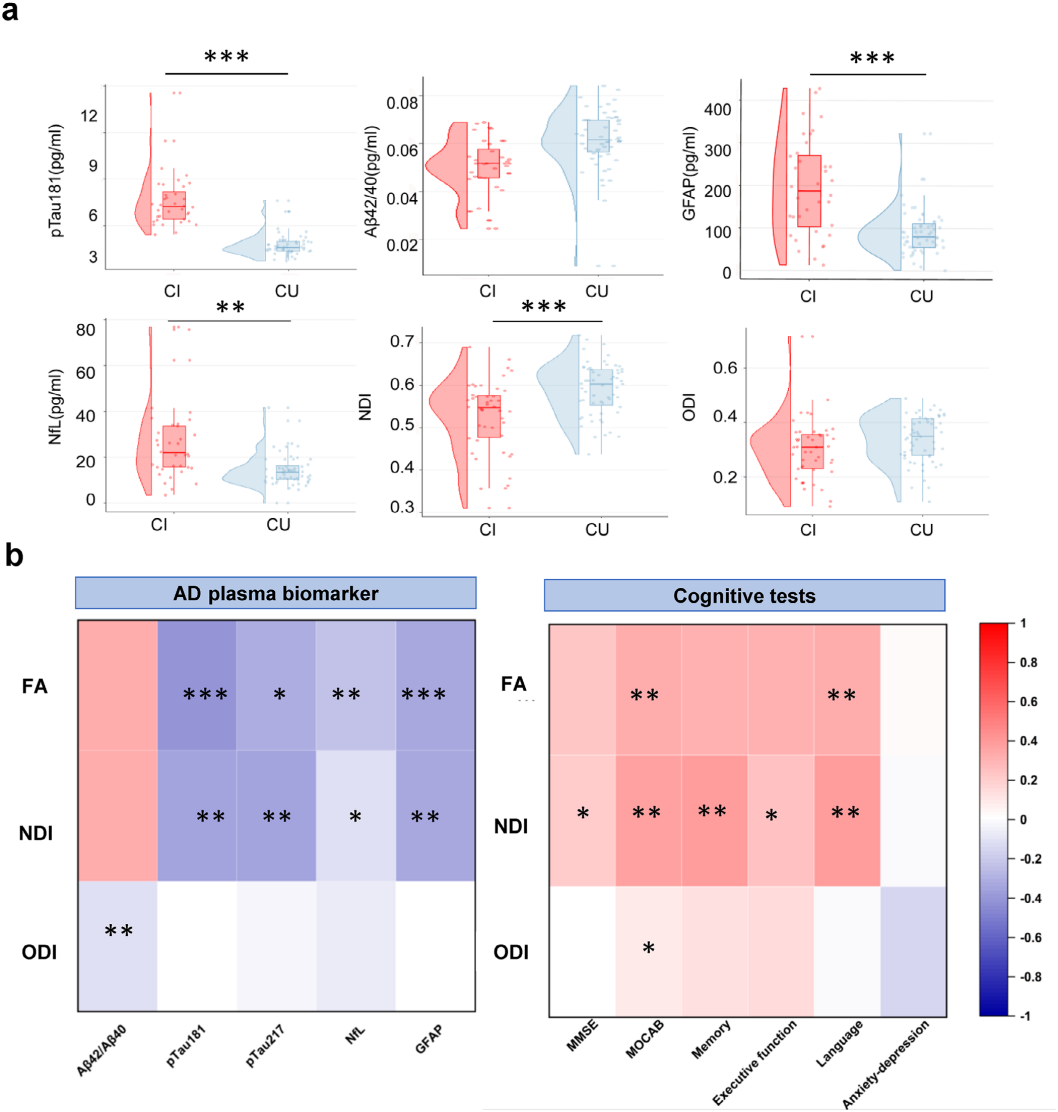# The relationship between white matter microstructure injury and plasma neuroinflammatory biomarkers and cognitive impairment : a NODDI and multi‐center study

**DOI:** 10.1002/alz70856_097595

**Published:** 2025-12-24

**Authors:** Xinru Xu, Bing Zhang

**Affiliations:** ^1^ Department of Radiology, Drum Tower Hospital, Clinical Colledge of Nanjing Medicial University, Nanjing 210008, china, Nanjing, Nanjing, China; ^2^ Nanjing Drum Tower Hospital, Nanjing, Jiangsu, China; ^3^ Department of Radiology, Nanjing Drum Tower Hospital, Affiliated Hospital of Medical School, Nanjing University, Nanjing, Jiangsu, China; ^4^ Nanjing Drum Tower Hospital, Affiliated Hospital of Medical School, Nanjing University, Nanjing, Jiangsu, China; ^5^ Nanjing Drum Tower Hospital Clinical College of Nanjing Medical University, Nanjing, China

## Abstract

**Background:**

Neurite Orientation Dispersion and Density Imaging (NODDI) is an advanced imaging technique capable of assessing synaptic density at the cellular level. Plasma markers such as Glial Fibrillary Acidic Protein (GFAP) and Neurofilament Light Chain (NfL) have recently been established as reliable indicators of neuroinflammation. However, the relationship between alterations in synaptic density within the white matter microstructure, residual neuroinflammation, and cognitive impairment in individuals with cognitive deficits remains poorly understood.

**Method:**

His study recruited 94 cognitively unimpaired (CU) individuals and 79 cognitively impaired (CI) participants, including patients with Alzheimer's disease (AD) and mild cognitive impairment (MCI). Each participant underwent Neurite Orientation Dispersion and Density Imaging (NODDI) and high‐resolution 3D structural MRI. Diffusion Tensor Imaging (DTI) metrics, such as fractional anisotropy (FA), and NODDI metrics, including the neurite density index (NDI) and orientation dispersion index (ODI), were compared between the CU and CI groups. Tract‐Based Spatial Statistics (TBSS) was used to evaluate microstructural alterations in the white matter (WM) fiber tracts of CI participants compared to healthy controls (HC).

**Result:**

Compared to the CU group, the FA, NDI, and ODI values of the white matter (WM) fiber bundles were significantly lower in CI patients. Fiber bundles, including the anterior thalamic radiation, cingulum, inferior fronto‐occipital fasciculus, corticospinal tract, and superior longitudinal fasciculus, were notably affected in patients with MCI and AD. NDI values in the anterior thalamic radiation, corticospinal tract, inferior fronto‐occipital fasciculus, and superior longitudinal fasciculus showed significant correlations with *p*‐tau181, NfL, GFAP, and cognitive function. The relationship between NDI and overall cognitive function exhibited an interactive effect between cognitively impaired and unimpaired individuals. NDI influences cognitive function by modulating pathways involving *p*‐tau181 and GFAP, but not through pathways involving Aβ42/40 and GFAP.

**Conclusion:**

The neurite density index (NDI), a key metric derived from NODDI (multi‐shell diffusion MRI), effectively captures neurodegenerative changes associated with Alzheimer's disease (AD) dementia. Neuroinflammation, as indicated by circulating GFAP levels, appears to impact cognitive function, particularly memory, by contributing to synaptic density loss in the white matter microstructure. These findings highlight neuroinflammation as a potential target for future disease‐modifying trials aimed at mitigating synaptic loss.